# Aerobic Exercise Ameliorates Myocardial Fibrosis via Affecting Vitamin D Receptor and Transforming Growth Factor-β1 Signaling in Vitamin D-Deficient Mice

**DOI:** 10.3390/nu15030741

**Published:** 2023-02-01

**Authors:** Xiaoning Cui, Ke Wang, Jinghua Zhang, Zhen-Bo Cao

**Affiliations:** School of Exercise and Health, Shanghai University of Sport, Shanghai 200438, China

**Keywords:** vitamin D deficiency, cardiovascular disease, aerobic exercise training, myocardial fibrosis

## Abstract

Myocardial fibrosis is a pathological phenomenon associated with cardiovascular disease (CVD) that plays a crucial role in the development of heart diseases. Vitamin D deficiency can promote the development of CVD and exercise plays a role in the treatment of CVD. This study aimed to explore the effects of 12-week aerobic exercise training on myocardial fibrosis in vitamin D-deficient mice. A vitamin D-deficient mouse model was induced by a vitamin D-deficient (0 IU Vitamin D_3_/kg) diet. Twenty-four C57BL/6J male mice were randomly divided into three groups: a control sedentary group (CONS, *n* = 8), a vitamin D-deficient sedentary group (VDDS, *n* = 8), and a vitamin D-deficient exercise group (VDDE, *n* = 8) which was aerobically trained for 12 weeks. The results showed that the serum 25-hydroxyvitamin D [25(OH)D] levels of the VDDS group were <50 nmol/L, which was significantly lower than that of the CONS group. Compared with the CONS group, the VDDS group showed cardiac dysfunction and significant fibrosis, together with lower vitamin D receptor (*VDR)* mRNA and protein expression levels, higher mRNA expression levels of profibrotic and inflammatory factors, and higher transforming growth factor-β1 (TGF-β1) and phospho-Smad2/3 (P-Smad2/3) protein expression levels. Serum 25(OH)D levels in the VDDE group were significantly higher than those in the VDDS group. Compared with the VDDS group, the VDDE group showed improved cardiac function and alleviated myocardial fibrosis. Meanwhile, the VDDE group had significantly higher *VDR* mRNA and protein expression levels; lower mRNA expression levels of profibrotic and inflammatory factors; and lower TGF-β1 and P-Smad2/3 protein expression levels. In conclusion, aerobic exercise training remains a promising intervention for treating myocardial fibrosis in vitamin D deficiency.

## 1. Introduction

Vitamin D deficiency is becoming an increasingly prevalent problem [1,2,3,4]. In 136 countries, 82.5% of 60,979 subjects had vitamin D deficiency or insufficiency, of which 26.4% of women and 18.4% of men had extreme vitamin D deficiency [5]. In China, the prevalence of vitamin D insufficiency and deficiency has reached 83% [6]. Even in Africa, with sufficient sunlight, the prevalence of vitamin D deficiency is still high [7]. Numerous epidemiological studies have shown that vitamin D deficiency may be associated with an elevated risk of multiple health problems [8], such as respiratory diseases [9], osteoporosis [10] and cardiovascular disease (CVD) [11,12].

CVD is a general term for all diseases related to the heart and blood vessels, and its scope covers a variety of diseases such as coronary heart disease, hypertension, and heart failure [13]. CVD is one of the many causes of death and chronic disability worldwide, and it has been reported that there were 523 million CVD events worldwide and approximately 18.6 million deaths in 2019 alone [14]. CVD is often accompanied by myocardial fibrosis, which plays an important role in disease development and progression [15]. Myocardial fibrosis can contribute to cardiac remodeling and dysfunction, resulting in heart failure [16]. Therefore, alleviating or reversing myocardial fibrosis represents an effective means of slowing the progression of CVD.

Individuals with vitamin D deficiency appear to be more susceptible to CVD and are significantly at risk for CVD [17,18,19,20]. In healthy adults, vitamin D deficiency and low 1,25(OH)_2_D_3_ levels are associated with increased arterial stiffness and vascular endothelial dysfunction, which are both predictors of CVD morbidity and mortality [21]. A 10-year follow-up study of 18,225 men found that low levels of 25-hydroxyvitamin D [25(OH)D] were still closely related to high-risk myocardial infarction even after controlling for confounding factors related to coronary heart disease [22]. A meta-analysis showed that the risk of death from CVD is increased by up to 57% in individuals with low 25(OH)D levels [23]. Lower vitamin D levels are significantly associated with a greater risk of CVD, and improvements to low vitamin D status in the population could effectively reduce the burden of CVD [24]. In addition, vitamin D deficiency has been associated with the severity of illness and poor prognostic outcomes in patients with CVD [25,26,27,28,29].

In animal studies, vitamin D deficiency has been found to contribute to the occurrence and development of myocardial fibrosis [30]. Vitamin D receptor (VDR) knockout mice have significantly larger left ventricular cardiomyocytes, higher cardiac atrial natriuretic peptide (*ANP*) mRNA expression [31] and higher levels of serum aldosterone and angiotensin II [32], all of which promote the development of myocardial fibrosis. The decreased expression of VDR is the representation of vitamin D deficiency, and the animal model of VDR knockout is also a method for studying vitamin D deficiency. VDR knockout mice with myocardial fibrosis have higher expression levels of matrix metalloproteinase-2 (*MMP-2*) and matrix metalloproteinase-9 (*MMP-9*) and lower expression levels of tissue inhibitors of metalloproteinase-1 (*TIMP-1*) [33]. This imbalance results in the deposition of the extracellular matrix (ECM) and the development of myocardial fibrosis. VDR overexpression significantly ameliorates myocardial fibrosis in pressure-overloaded hearts and decreases mRNA expression levels of collagen I (*Col1*), collagen III (*Col3*), and *TIMP-1* [34]. Notably, 1,25(OH)_2_D_3_ and calcitriol supplementation ameliorates myocardial fibrosis by enhancing VDR proteins [35,36,37]. The above studies suggest that VDR regulates ECM metabolism in cardiomyocytes, which plays a role in regulating myocardial fibrosis.

Vitamin D can affect the physiological function of the body, mainly by forming the hormone–receptor complex with VDR in target cells. Subsequently, the complex is transported to the nucleus and then combined with the promoter region of the target gene to regulate gene expression [38]. VDR is a nuclear hormone receptor, which exists in most functional organs of the body, especially in the heart and blood vessels [37,39]. In the cardiovascular system, the deletion of VDR is associated with adverse cardiac remodeling, hypertrophy, and inflammatory response [34,40]. While the activation of VDR can improve cardiac dysfunction, it can also alleviate myocardial fibrosis and inflammatory responses [35,37,40,41]. These results suggest that VDR may be a potential target for the treatment of CVD.

An increasing number of studies have shown that exercise training is an effective strategy to alleviate myocardial fibrosis [42,43,44,45,46]. Studies have found that aerobic exercise training reduces myocardial fibrosis by inhibiting the transforming growth factor-β1 (TGF-β1)-Smad2/3 signaling pathway and decreasing Col1 and Col3 protein expression in myocardial infarcted rats [43,47]. As a key growth factor that regulates the fibrotic process, the activity of TGF-β is significantly increased after myocardial injury and participates in the regulation of the phenotype and function of all cells that promote myocardial fibrosis. TGF-β can stimulate the proliferation and differentiation of cardiac fibroblasts while enhancing the synthesis of ECM proteins. TGF-β also regulates homeostasis between MMPs and TIMPs to regulate ECM synthesis and catabolic pathways [48,49]. Both in vitro and in vivo evidence suggests that elevated TGF-β1 levels activate the downstream Smad2/3 pathway, thereby promoting the process of myocardial fibrosis [50,51].

Many studies have found that vitamin D deficiency is evident in most patients with CVD [52,53,54,55,56]. Although aerobic exercise training is an effective therapy for the prevention and treatment of CVD [57,58,59,60], its effects are not clear when the body is vitamin D deficient. One study found that aerobic exercise training while vitamin D deficient failed to counter certain metabolic syndrome indicators [61], suggesting that adequate vitamin D status is required to enable the beneficial effects of exercise. However, another study also showed that swimming improved serum 25(OH)D levels and the expression of VDR in some tissues of diabetic rats with vitamin D deficiency, thereby exerting the beneficial effects of exercise [62]. Therefore, the purpose of our study was to explore whether aerobic exercise training could alleviate myocardial fibrosis in patients with vitamin D deficiency. We hypothesized that aerobic exercise training could alleviate myocardial fibrosis and enhance cardiac function in vitamin D-deficient mice, possibly by increasing VDR expression and inhibiting the TGF-β1-Smad2/3 pathway.

## 2. Materials and Methods

### 2.1. Experimental Animals

When studying the development of heart disease, the choice of a suitable animal model should be considered carefully. Because of the high homology between mouse gene and human gene, mouse has become the primary animal choice for studying human heart disease [63]. Twenty-four 6-week-old C57BL/6J male mice were purchased from GemPharmatech (Nanjing, China). All operations were conducted in accordance with the relevant guidelines and regulations of the Ethics Committee of the Shanghai University of Sport (No. 102772021DW008). All mice were housed in a specific-pathogen-free grade animal room with a 12:12 hr light-dark cycle (lights on at 08:30, lights off at 20:30), temperature of 22 ± 2 °C, and relative humidity of 50–70%. Yellow light was used throughout this experiment to prevent mice from synthesizing vitamin D. The mice were allowed to eat and drink freely throughout the experiment, except during fasting.

Twenty-four mice were randomly divided into three groups: control sedentary group (CONS, *n* = 8), vitamin D-deficient sedentary group (VDDS, *n* = 8), and vitamin D-deficient exercise group (VDDE, *n* = 8). Feed for the mice was customized and purchased from Jiangsu Synergy Bio (Nanjing, China). The feed composition of the CONS group was mainly 20.3 kcal% protein, 63.95 kcal% carbohydrates, 15.75 kcal% fat and 1000 IU vitamin D_3_/kg (VD_3_/kg). The feed compositions of VDDS and VDDE groups were mainly 20.3 kcal% protein, 63.95 kcal% carbohydrates, 15.75 kcal% fat and 0 IU VD_3_/kg. The first week to the twelfth week was termed the modeling period. At the end of the modeling period, serum 25(OH)D levels were measured in each group to determine whether a model of vitamin D deficiency in mice had been successfully established. After successful modeling, the VDDE group began aerobic exercise training, and the other two groups were free to move around. The intervention period was from the first week to the twelfth week after formal exercise. At the end of the intervention period, cardiac function was measured using echocardiography, and serum samples and heart tissues were collected and stored at −80 °C for further analysis.

### 2.2. Exercise Program

After one week of exercise pre-adaptation, mice in the VDDE group were subjected to moderate-intensity aerobic exercise training with increasing loads on a running platform with an incline of 0°. The exercise training protocol (Table S1) was adjusted based on previous studies [64]. Training was performed for 12 weeks, six days per week, and 60 min per day. The mice were warmed and relaxed for 10 min before and after exercise.

### 2.3. Body Weight and Body Composition

During this experiment, the weight of the mice was measured weekly. Before sacrificing the mice, lean mass and body fat rate were measured using an Echo MRI animal body composition analyzer (San Diego, CA, USA).

### 2.4. Echocardiography

To reduce subjectivity, an experienced technician who was blinded to the purpose and content of this study conducted testing. Before sacrificing the mice, the technician used a small-animal ultrasound imaging system (VisualSonics, Toronto, ON, Canada, Vevo 2100) to measure cardiac function. The indices obtained were as follows: left ventricular internal dimension at systole (LVIDs), left ventricular posterior wall at systole (LVPWs), interventricular septal thickness at systole (IVSs), left ventricular internal dimension at diastole (LVIDd), left ventricular posterior wall at diastole (LVPWd), and interventricular septal thickness at diastole (IVSd). The ejection fraction (EF) and fraction shortening (FS) of the left ventricle were calculated using the following formulae:

EF = [(LVIDd)^3^ − (LVIDs)^3^)/(LVIDd)^3^] × 100%, FS = [(LVIDd − LVIDs)/LVIDd] × 100%.

### 2.5. Serum Biochemical Testing

Serum indices in mice were measured using the following ELISA kits: 25(OH)D (Immunodiagnosticsystems, Boldon, UK, AC-57SF1), parathyroid hormone (PTH, Nanjing Jiancheng Bioengineering Institute, Nanjing, China, H207), and insulin (INS, Nanjing Jiancheng Bioengineering Institute, Nanjing, China, H203-1-2). Kits used to measure other serum indices were as follows: calcium (Ca, Nanjing Jiancheng Bioengineering Institute, Nanjing, China, C004-2-1), phosphorus (P, Nanjing Jiancheng Bioengineering Institute, Nanjing, China, C006-1-1), low-density lipoprotein cholesterol (LDL-C, Nanjing Jiancheng Bioengineering Institute, Nanjing, China, A113-1-1), high-density lipoprotein cholesterol (HDL-C, Nanjing Jiancheng Bioengineering Institute, Nanjing, China, A112-1-1), total cholesterol (TC, Nanjing Jiancheng Bioengineering Institute, Nanjing, China, A111-1-1), and triacylglycerol (TG, Nanjing Jiancheng Bioengineering Institute, Nanjing, China, A110-1-1).

### 2.6. Morphology

Heart tissues were immersed in 4% paraformaldehyde for 48 hr before routine paraffin embedding. Paraffin wax blocks were cut into 5 μm sections for hematoxylin and eosin (H&E), wheat germ agglutinin (WGA), and Sirius red (SR) staining to observe the morphology, cross-sectional area (CSA), and degree of fibrosis of cardiomyocytes.

### 2.7. Quantitative Real-Time PCR

Heart tissue (40 mg) was weighed and placed in an Eppendorf (EP) tube, and 1 mL of total RNA extraction reagent (TRIzol) was added. After grinding and centrifugation, the supernatant was extracted, and chloroform was added. After thorough mixing, the mixture was centrifuged and the supernatant was added to an equal volume of isopropanol solution and centrifuged again to obtain an RNA precipitate. Then, diethylpyrocarbonate (DEPC) water was added to dissolve the RNA precipitate and the level and purity of RNA were checked.

The reaction system was assembled according to the manufacturer’s instructions (Thermo Fisher Scientific, Waltham, MA, USA, K1622), and the RNA was reverse-transcribed into cDNA. Primer sequences of target genes were obtained from the PubMed website (Table S2). An appropriate amount of Tris-EDTA buffer was added to dissolve the primers. The upstream and downstream PCR primers, DEPC-treated water, and master mix were added to an EP tube and the mixture was fully mixed. Notably, 18 µL of mixture and 2 µL of sample were added to each well of a 96-well plate. The amplification program was as follows: 95 °C for 30 sec, 40 cycles of 95 °C for 10 sec and 60 °C for 30 sec, and finally 95 °C for 15 sec, 60 °C for 60 sec and 95 °C for 15 sec. GAPDH was used as an internal reference, and relative expression levels of the target genes in each group were calculated using formula.

### 2.8. Western Blotting

Heart samples (20 mg) were lysed in a mixture of radioimmunoprecipitation assay lysate (Beyotime, Shanghai, China, P0013B) and phenylmethanesulfonyl fluoride (Beyotime, Shanghai, China, ST506). After centrifugation, the supernatant was extracted from the mixture and placed in an EP tube. Protein levels in the samples were detected using a kit (Beyotime, Shanghai, China, P0012S). After testing, the protein samples were uniformly diluted 10-fold and boiled in a metal bath at 100 °C for 10 min to denature the protein. The separation and spacer gels were prepared in advance according to the molecular weight of the target protein. After gel preparation, 40 µg of protein sample or 2 µL of marker was added to each lane to start electrophoresis and membrane transfer, ensuring protein transfer to 0.22 μm or 0.44 μm polyvinylidene difluoride membranes (Millipore, Bedford, MA, USA). After sufficient washing with tris-buffered saline with Tween 20 (TBST), the membranes were placed in 5% skim milk and incubated for 2 hr. Afterwards, the membranes were washed with TBST and incubated overnight at 4 °C with primary antibodies against TGF-β1 (1:500, Abcam, Cambridge, UK, ab92486), Smad2/3 (1:1000, Cell Signaling Technology, Boston, MA, USA, 8685S), phospho-Smad2/3 (P-Smad2/3, 1:1000, Cell Signaling Technology, 8828), VDR (1:500, Santa Cruz Biotechnology, Dallas, TX, USA, 13133), and GAPDH (1:5000, Proteintech, Wuhan, China, 60004-1-lg). TBST was used to wash away unbound primary antibodies, and the membranes were incubated with secondary antibodies (Absin, Shanghai, China) at room temperature for 1.5 hr. After preparing electrochemiluminescence solution (Millipore), the membranes were washed with TBST and placed under an imager for development and photography. Grayscale values of the target proteins were calculated using Image J 1.53k software (National Institutes of Health, Bethesda, MD, USA).

### 2.9. Statistical Analysis

The data obtained in experiments were summarized, and SPSS 26.0 (International Business Machines Corporation, Amunk, NY, USA) was used for experimental data processing and analysis. Data satisfying a normal distribution were expressed as means ± standard deviation, while data not satisfying a normal distribution were expressed as medians (quartile distance). Data were analyzed using one-way ANOVA (normal distribution) or the Kruskal-Wallis test (non-normal distribution). Differences between the groups were tested using the Bonferroni post hoc test, and *p* < 0.05 was considered to be statistically significant.

## 3. Results

### 3.1. The Modeling of Vitamin D-Deficient Mice

**Figure 1:**
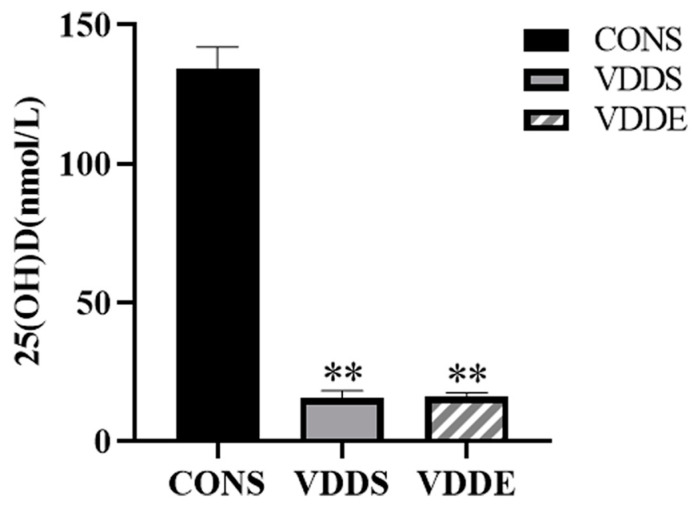
Serum 25(OH)D levels in mice after the modeling period. *n* = 5; ** *p* < 0.01 vs. CONS group.

At the end of the modeling period, serum 25(OH)D levels in the VDDS and VDDE groups were significantly lower than those in the CONS group (*p* < 0.01) and were <50 nmol/L, demonstrating the successful modeling of vitamin D-deficient mice (Figure 1).

### 3.2. Effect of Aerobic Exercise Training on the Serum 25(OH)D Levels, Body Weight, Lean Mass and Body Fat Rate of Vitamin D-Deficient Mice

**Figure 2:**
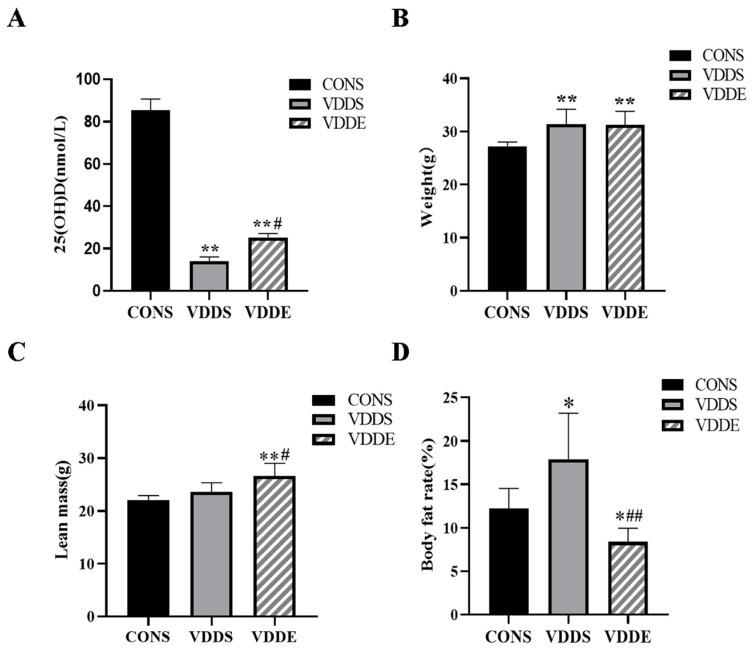
Serum 25(OH)D levels (**A**), body weight (**B**), lean mass (**C**) and body fat rate (**D**) of mice after the intervention period. *n* = 8; * *p* < 0.05 vs. CONS group, ** *p* < 0.01 vs. CONS group; # *p* < 0.05 vs. VDDS group, ## *p* < 0.01 vs. VDDS group.

At the end of the intervention period, serum 25(OH)D levels of the VDDS and VDDE groups were significantly lower than those of the CONS group (*p* < 0.01), and serum 25(OH)D levels of the VDDE group were significantly higher than in the VDDS group (*p* < 0.05) (Figure 2A). The body weights of the VDDS and VDDE group animals were significantly higher than those of the CONS group (*p* < 0.01) (Figure 2B). The lean mass of the VDDE group was significantly higher than those of the CONS group and VDDE group (*p* < 0.01, *p* < 0.05) (Figure 2C). Compared with the CONS group, the body fat rate of the VDDS group was significantly higher (*p* < 0.05) and that of the VDDE group was significantly lower (*p* < 0.05). Compared with the VDDS group, the body fat rate in the VDDE group was also significantly lower (*p* < 0.01) (Figure 2D).

### 3.3. Aerobic Exercise Training Caused Myocardial Hypertrophy in Vitamin D-Deficient Mice

There was no significant difference in heart weight/tibial length (HW/TL) and left ventricle weight/tibial length (LVW/TL) between the CONS and VDDS groups (*p* > 0.05). The HW/TL and LVW/TL of the VDDE group were significantly greater than those of the CONS (*p* < 0.05, *p* < 0.01) and VDDS groups (*p* < 0.05) (Table 1), suggesting that aerobic exercise training still caused myocardial hypertrophy while vitamin D-deficient. However, there was no significant difference in serum indices of all the groups (*p* > 0.05) (Table 2).

### 3.4. Aerobic Exercise Training Improved Cardiac Function in Vitamin D-Deficient Mice

**Figure 3:**
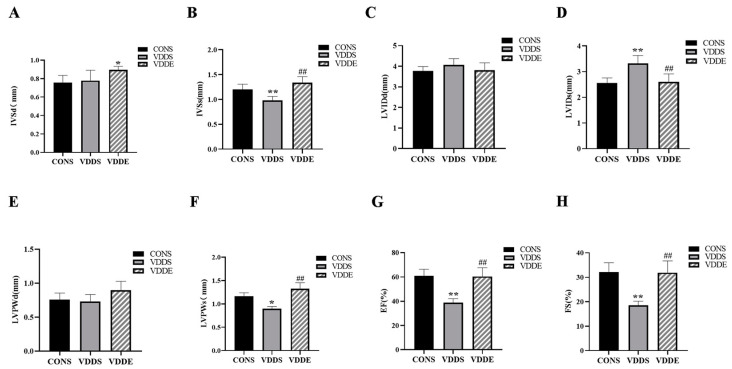
Heart ultrasound-related data in mice after the intervention period. (**A**) IVSd, (**B**) IVSs, (**C**) LVIDd, (**D**) LVIDs, (**E**) LVPWd, (**F**) LVPWs, (**G**) EF, (**H**) FS. *n* = 6; * *p* < 0.05 vs. CONS group, ** *p* < 0.01 vs. CONS group; ## *p* < 0.01 vs. VDDS group.

Compared with the CONS group, LVIDs was significantly larger (*p* < 0.01) (Figure 3D), and IVSs (*p* < 0.01), LVPWs (*p* < 0.05), EF (*p* < 0.01) and FS (*p* < 0.01) were significantly smaller in the VDDS group (Figure 3B,F–H). The IVSd (*p* < 0.05) was significantly larger in the VDDE group than in the CONS group (Figure 3A). LVIDs was significantly smaller (*p* < 0.01) (Figure 3D) and IVSs, LVPWs, EF, and FS were significantly larger (*p* < 0.01) in the VDDE group than in the VDDS group (Figure 3B,F–H), suggesting that aerobic exercise training could improve cardiac function in vitamin D-deficient mice. However, there was no significant difference in LVIDd and LVPWd between the groups (*p* > 0.05) (Figure 3C,E).

### 3.5. Effect of Aerobic Exercise Training on the Myocardial Morphology of Vitamin D-Deficient Mice

**Figure 4:**
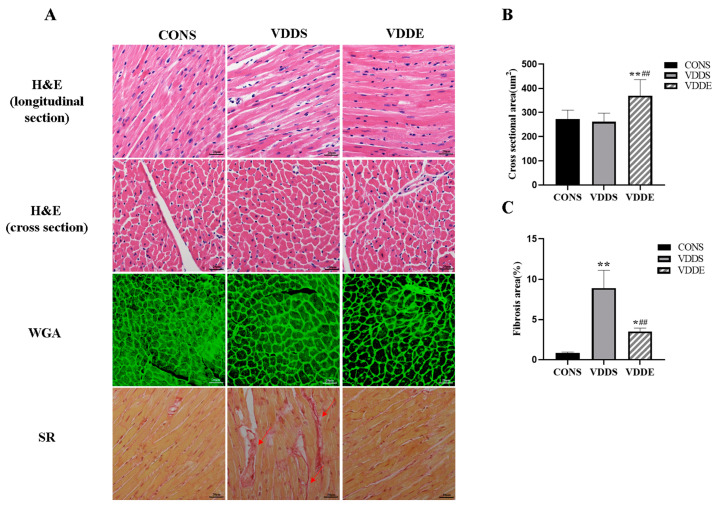
H&E, WGA, and SR staining and statistical results for left ventricular myocardium after the intervention period. *n* = 3; (**A**) H&E, WGA, and SR staining of the left ventricular myocardium, ×400, Bar = 20 μm, the red arrows indicate areas of fibrosis, (**B**) Myocardial CSA statistics, and (**C**) SR staining statistics. * *p* < 0.05 vs. CONS group, ** *p* < 0.01 vs. CONS group, ## *p* < 0.01 vs. VDDS group.

H&E staining results showed that the myocardium in the CONS group was more closely arranged with a normal cell structure, while the myocardial gap of the VDDS group was significantly enlarged, and some myocardial fibers were broken and lysed. The myocardial gap in the VDDE group was significantly reduced, the myocardium was more densely arranged, and the cell structure was nearly normal. From a cross-sectional view, H&E and WGA staining showed that myocardial cells of the VDDS group were not significantly hypertrophic compared with those of the CONS group, while mice in the VDDE group exhibited significant myocardial hypertrophy (Figure 4A). Statistical results showed that the myocardial CSA of the VDDE group was significantly larger than that of the CONS and VDDS groups (*p* < 0.01) (Figure 4B). SR staining and statistical results showed that the myocardium of the VDDS group showed significant fibrosis compared to the CONS group (*p* < 0.01), and the degree of myocardial fibrosis was significantly alleviated in the VDDE group compared to the VDDS group (*p* < 0.01) (Figure 4A,C). These results showed that aerobic exercise training could alleviate myocardial fibrosis caused by vitamin D deficiency.

### 3.6. Aerobic Exercise Training Upregulated Myocardial VDR Expression in Vitamin D-Deficient Mice

**Figure 5:**
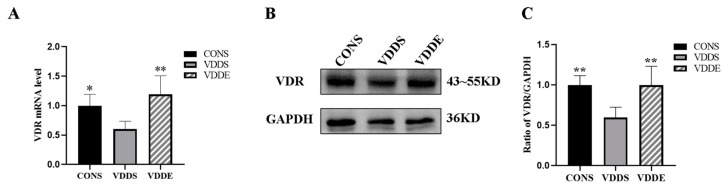
Effects on myocardial VDR after the intervention period. *n* = 6; (**A**) mRNA expression of *VDR*, (**B**) protein expression of VDR, and (**C**) densitometry analysis of VDR protein expression. * *p* < 0.05 vs. VDDS group, ** *p* < 0.01 vs. VDDS group.

*VDR* mRNA expression was significantly higher in the CONS (*p* < 0.05) and VDDE (*p* < 0.01) groups than in the VDDS group. Western blotting results showed that VDR protein expression was significantly higher in the CONS and VDDE groups than in the VDDS group (*p* < 0.01) (Figure 5).

### 3.7. Aerobic Exercise Training Downregulated Myocardial Fibrotic Factors Expression in Vitamin D-Deficient Mice

**Figure 6:**
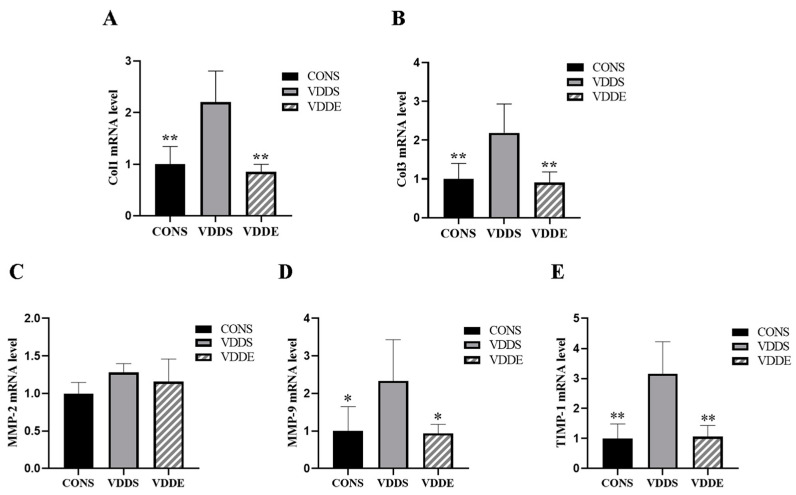
mRNA expression of myocardial fibrotic factors after the intervention period. *n* = 6; (**A**) *Col1*, (**B**) *Col3*, (**C**) *MMP-2*, (**D**) *MMP-9*, and (**E**) *TIMP-1* expression. * *p* < 0.05 vs. VDDS group, ** *p* < 0.01 vs. VDDS group.

The mRNA expression of *Col1* (*p* < 0.01), *Col3* (*p* < 0.01), *MMP-9* (*p* < 0.05), and *TIMP-1* (*p* < 0.01) was significantly lower in the VDDE and CONS groups than in the VDDS group (Figure 6A,B,D,E), suggesting that aerobic exercise training reduced collagen deposition in the myocardium of vitamin D-deficient mice. There was no significant difference in the mRNA expression of *MMP-2* between the groups (*p* > 0.05) (Figure 6C).

### 3.8. Aerobic Exercise Training Inhibited TGF-β1-Smad2/3 Pathway in Vitamin D-Deficient Mice

**Figure 7:**
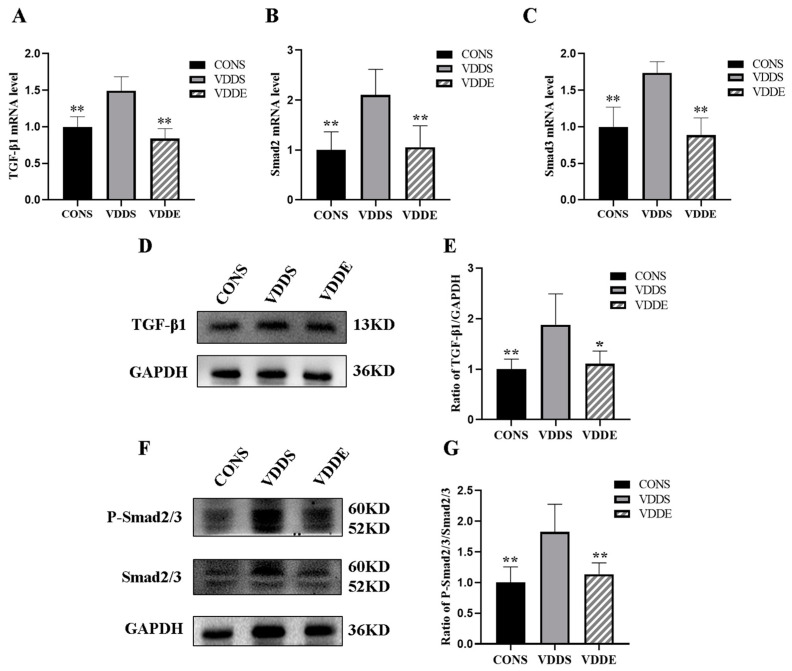
Effects on myocardial TGF-β1 and Smad2/3 after the intervention period. *n* = 6; (**A**) mRNA expression of *TGF-β1*, (**B**) mRNA expression of *Smad2*, (**C**) mRNA expression of *Smad3*, (**D**) Protein expression of TGF-β1, (**E**) Densitometry analysis of TGF-β1 protein expression, (**F**) Protein expression of P-Smad2/3, (**G**) Densitometry analysis of P-Smad2/3 protein expression. * *p* < 0.05 vs. VDDS group, ** *p* < 0.01 vs. VDDS group.

The mRNA expression levels of *TGF-β1*, *Smad2*, and *Smad3* were significantly lower in the VDDE group and CONS group than in the VDDS group (*p* < 0.01) (Figure 7A–C), indicating that aerobic exercise training could downregulate the expression of profibrotic factors in vitamin D-deficient mice. The expression of TGF-β1 (*p* < 0.05, *p* < 0.01) and P-Smad2/3 (*p* < 0.01) proteins was significantly lower in the VDDE group and CONS group than in the VDDS group (Figure 7D–G), suggesting that aerobic exercise training may ameliorate myocardial fibrosis in vitamin D-deficient mice by inhibiting the TGF-β1-Smad2/3 signaling pathway.

### 3.9. Aerobic Exercise Training Downregulated Inflammatory Factors Expression in Vitamin D-Deficient Mice

**Figure 8:**
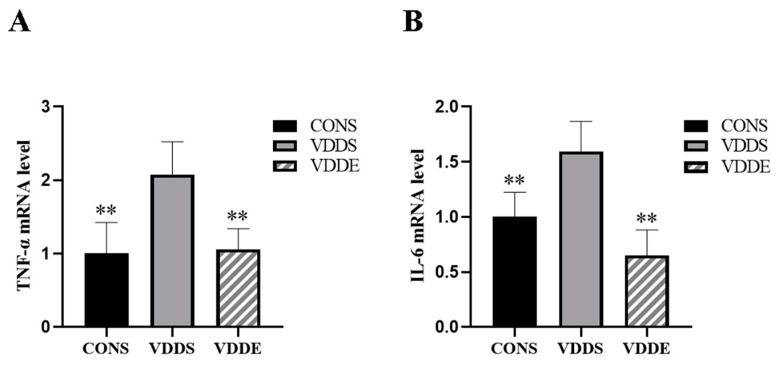
mRNA expression of *TNF-α* (**A**) and *IL-6* (**B**) after the intervention period. *n* = 6; ** *p* < 0.01 vs. VDDS group.

As shown in Figure 8, the mRNA expression levels of tumor necrosis factor alpha (*TNF-α*) and interleukin-6 (*IL-6*) were significantly lower in the VDDE group and CONS group than in the VDDS group (*p* < 0.01), indicating that aerobic exercise training could improve inflammatory response in vitamin D-deficient mice.

## 4. Discussion

C57BL/6J male mice were used to generate an animal model of vitamin D-deficient mice through dietary intervention. After successful modeling, mice in the VDDE group underwent 12 weeks of moderate-intensity aerobic exercise training. We observed that vitamin D deficiency induced cardiac dysfunction, myocardial fibrosis, and inflammation in mice, while aerobic exercise training improved cardiac dysfunction and alleviated the progression of myocardial fibrosis and inflammation in vitamin D-deficient mice.

To improve the success rate of modeling, mice in the VDDS and VDDE groups were fed no vitamin D_3_-containing food (0 IU VD_3_/kg) for 12 weeks [65,66]. Yellow lamps were used during the experiment to eliminate all potential ultraviolet light sources, thus minimizing the synthesis of endogenous vitamin D in the skin [67]. The above methods could provide a sufficient guarantee for the modeling of vitamin D deficiency in mice. The level of serum 25(OH)D was used to assess the nutritional status of vitamin D. Vitamin D deficiency and insufficiency were defined by serum 25(OH)D levels <20 ng/mL (50 nmol/L) and 20–30 ng/mL (50–75 nmol/L), respectively, and only levels >30 ng/mL (>75 nmol/L) were considered vitamin D-sufficient [68]. Serum 25(OH)D levels in the VDDS and VDDE groups were <50 nmol/L, which was significantly lower than that in the CONS group. Therefore, we concluded that an animal model of vitamin D-deficient mice was successfully constructed.

According to SR staining results, vitamin D deficiency caused myocardial interstitial fibrosis in the VDDS group. The production of fibrosis is mainly related to the deposition of ECM, and collagen plays an important role in this process, involving *Col1* and *Col3*. It was found that the mRNA and protein expression of *Col1* and *Col3* were significantly increased in heart tissue where fibrosis occurred [69]. In this study, the mRNA expression levels of *Col1* and *Col3* were significantly higher in the VDDS group than in the CONS group. In addition, an imbalance between MMPs and TIMPs plays a significant role in the progression of myocardial fibrosis. In previous studies, the expression of *MMP-2* and *MMP-9* increased in the left ventricular myocardium in patients with dilated cardiomyopathy [70], and the activation of *MMP-2* and *MMP-9* occurred in the myocardium of mice with heart failure, accompanied by extensive collagen deposition degeneration [71]. Collagen deposition in the myocardium is accompanied by MMP activation, which is closely related to the process of ventricular remodeling. Inhibition of MMP activity can reduce the degree of myocardial fibrosis and hypertrophy [72,73]. Researchers have found increased collagen deposition in VDR-knockout mice, which was associated with increased *MMP-2* and *MMP-9* activity and decreased *TIMP-1* activity [33]. The results of this study showed that the expression levels of *MMP-9* and *TIMP-1* were significantly increased in the VDDS group compared to the CONS group, and the increased expression level of *TIMP-1* may be a response to *MMP-9* overactivation. TGF-β1 is a growth factor that plays an important role in the fibrotic process and is hyperactivated in remodeled and fibrotic hearts. Numerous studies have shown that the appearance of fibrosis in the myocardium is associated with the activation of the TGF-β1-Smad2/3 pathway [74,75]. Compared with the CONS group, the VDDS group had significantly lower mRNA and protein expression levels of VDR, activation of the TGF-β1-Smad2/3 signaling pathway, and increased mRNA expression of profibrotic factors (*Col1*, *Col3*, *MMP-9*, *TIMP-1*). Since inflammation is involved in the development of tissue fibrosis [76], we detected the mRNA expression of related inflammatory factors. The VDDS group had significantly higher expression of *TNF-α* and *IL-6* than the CONS group. Thus, we speculated that vitamin D deficiency might induce myocardial fibrosis by inhibiting VDR, activating the TGF-β1-Smad2/3 pathway, and inducing inflammatory infiltration.

We observed that serum 25(OH)D levels were significantly higher in the VDDE group than in the VDDS group, indicating that aerobic exercise training increased serum 25(OH)D levels in vitamin D-deficient mice. Consistently, our previous research also demonstrated an improvement effect of exercise on serum 25(OH)D levels [77,78]. There was no significant difference between the VDDS and VDDE groups in other serum indices, such as Ca, P, PTH, TC, and LDL-C. We suspect that the absent effects of exercise might be related to the unchanged serum indices in the VDDS group, or that its effects were offset by vitamin D deficiency [61].

The increase in heart weight was mainly related to an increase in myocardial CSA. An increasing number of studies have shown that exercise can induce physiological hypertrophy of the heart [79,80,81]. Physiologically, hypertrophic hearts maintain or increase systolic function without cell death, while pathologically hypertrophic hearts are often accompanied by cardiomyocyte death, myocardial fibrosis, and systolic dysfunction, which will further progress to heart failure [82]. In this study, we found that aerobic exercise training significantly increased HW/TL, LVW/TL, and CSA of the left ventricle myocardium in the VDDE group. The IVSs and LVPWs were also larger. These results suggested that aerobic exercise training could promote left ventricle hypertrophy even in vitamin D-deficient mice, extending the existing findings to a model of vitamin D-deficient mice for the first time. At the same time, we observed a significantly larger EF and FS in the VDDE group, which demonstrated an improvement in cardiac function. These results suggested that aerobic exercise training induced physiologic hypertrophy and played an important role in cardiac remodeling and functional improvement in vitamin D-deficient mice.

In terms of myocardial morphology, aerobic exercise training improved the structure of cardiomyocytes by reducing myocardial gap and alleviated the degree of myocardial fibrosis in VDDE mice. Meanwhile, the mRNA and protein expression levels of VDR were significantly increased in the VDDE group, accompanied by inhibition of the TGF-β1-Smad2/3 signaling pathway. In addition, decreased mRNA expression of profibrotic factors (*Col1*, *Col3*, *MMP-9*, and *TIMP-1*) and inflammatory factors (*TNF-α* and *IL-6*) were found in the VDDE group. Based on the above results, aerobic exercise training may have exerted its effects by increasing the expression of VDR in this study. Aerobic exercise training could be an effective intervention strategy to ameliorate myocardial fibrosis and improve cardiac function in patients with vitamin D deficiency. This discovery can provide a scientific basis for the treatment of patients suffering from vitamin D deficiency.

However, our study had certain limitations. This study failed to include a vitamin D supplementation group. Perhaps vitamin D supplementation may be more effective than aerobic exercise training. Whether aerobic exercise training combined with vitamin D supplementation exerts a combined effect remains to be explored in the future [83]. This study explored only one of several signaling pathways, more studies are needed in the future to explore other signaling pathways. In addition, VDR knockout mice should be used to reinforce the above results and further explore the molecular mechanisms.

## 5. Conclusions

In this study, we found that vitamin D deficiency impaired cardiac contractile function in mice, accompanied by myocardial fibrosis and inflammation. Myocardial fibrosis induced by vitamin D deficiency may be associated with decreased VDR expression, activation of the TGF-β1-Smad2/3 signaling pathway, and increased expression of profibrotic and inflammatory factors. This study demonstrated for the first time that aerobic exercise training could alleviate myocardial fibrosis in vitamin D-deficient mice, which may be associated with increased VDR expression, inhibition of the TGF-β1-Smad2/3 pathway, and decreased expression of profibrotic and inflammatory factors.

## Figures and Tables

**Table 1:** HW/TL and LVW/TL in mice after the intervention period.

	CONS	VDDS	VDDE
HW/TL (mg/mm)	7.44 ± 0.61	7.65 ± 0.72	8.65 ± 0.85 *#
LVW/TL (mg/mm)	2.87 ± 0.34	3.00 ± 0.19	3.48 ± 0.38 **#

All data in the table are mean ± standard deviation. *n* = 8; * *p* < 0.05 vs. CONS group, ** *p* < 0.01 vs. CONS group; # *p* < 0.05 vs. VDDS group.

**Table 2:** Serum indices in mice after the intervention period.

	CONS	VDDS	VDDE
Ca (mmol/L)	1.68 (1.63, 1.70)	1.67 ± 0.04	1.66 (1.56, 1.69)
P (mmol/L)	1.97 ± 0.46	2.15 ± 0.33	2.26 ± 0.32
HDL-C (mmol/L)	4.10 ± 0.47	3.74 ± 0.50	3.84 ± 0.21
LDL-C (mmol/L)	1.18 ± 0.44	0.87 ± 0.64	1.32 ± 0.43
TG (mmol/L)	1.12 ± 0.17	1.28 ± 0.17	1.14 ± 0.16
TC (mmol/L)	2.65 ± 0.42	2.71 ± 0.30	2.81 ± 0.33
PTH (ng/L)	210.52 ± 51.03	178.19 ± 50.39	225.74 ± 30.16
INS (ng/L)	22.40 ± 4.27	22.96 ± 7.37	21.32 ± 4.85

All data are presented as mean ± standard deviation or median (interquartile range). *n* = 6.

## Data Availability

The data that support the findings of this study are available from the corresponding author upon reasonable request.

## Supplementary Materials

The following supporting information can be downloaded at: https://www.mdpi.com/article/10.3390/nu15030741/s1, Table S1: Aerobic exercise protocol; Table S2: Sequence primers.
