# Supplementary material for: Aerobic Exercise Ameliorates Myocardial Fibrosis via Affecting Vitamin D Receptor and Transforming Growth Factor-β1 Signaling in Vitamin D-Deficient Mice
Source: Nutrients. 2023 Feb 1;15(3):741. doi: 10.3390/nu15030741 (PMC9919278; doi:10.3390/nu15030741)
Supplement: Supplementary file 1 [file nutrients-15-00741-s001.zip › nutrients-2162222-supplementary.pdf]

Supplementary Materials:

Table S1. Aerobic exercise protocol.

Training week	Speed(m/min)	Time(min)
1	10	60
2	11	60
3	12	60
4	13	60
5	14	60
6	14	60
7	15	60
8	15	60
9	16	60
10	16	60
11	17	60
12	17	60

Table S2. Sequence primers.

Target gene	Forward	Reverse
Collagen I	AGTCGATGGCTGCTCCAAAA	AGCACCACCAATGTCCAGAG
Collagen III	TCCTGGTGGTCCTGGTACTG	AGGAGAACCACTGTTGCCTG
TGF-β1	CTAATGGTGGACCGCAACAAC	CACTGCTTCCCGAATGTCTGA
Smad 2	AGCAGGAATTGAGCCACAGAGT	GACAGGGGAGAGAGTGGTAGGAG
Smad 3	GAGCTTACAAGGCGGCACATT	GGGAGACTGGACGAAAATAGCA
MMP-2	CCTTCACTTTCCTGGGCAAC	GGTGTAGGTGTAGATCGGGG
MMP-9	CGTCGTGATCCCCACTTACT	AACACACAGGGTTTGCCTTC
TIMP-1	GCAACTCGGACCTGGTCATAA	CGGCCCGTGATGAGAAACT
TNF-α	CCACGCTCTTCTGTCTACTGA	AAGGTACAACCCATCGGCTG
IL-6	ACAACCACGGCCTTCCCTACTT	CACGATTTCCCAGAGAACATGTG
VDR	GTGGACATTGGCATGATGAA	TTACGTCTGCACGAATTGGA
GAPDH	ACTCCACTCACGGCAAATTC	TCTCCATGGTGGTGAAGACA
